# The Chief Adornment

**Published:** 1875-05

**Authors:** 


### THE CHIEF ADORNMENT.

Of all the ornaments of the human face, the teeth stand pre-eminent. Such a thing as a truly attractive face, with decayed, deformed or missing teeth, was never seen. On the other hand, the most irregular features, and the most dingy complexion, are essentially redeemed and beautified by perfect teeth.

These are simple truths, known of all men and women. They embody no new fact. They are not peculiar to any nation or period. They are found in recorded writings of all lands and of every age of the world. Before printing was known, before literature, worthy of the name, was born, these and kindred thoughts were among the utterances of the wisest philosophers. Our Bible is not without allusions to the beauty of the teeth, and the sacred writings of all peoples assert the value of the dental structure. The Persian Bible declares that the teeth are more valuable than pearls ; and the Talmud places them above diamonds.

If, then, they are so valuable, should they not be preserved in all their beauty and usefulness ? Hall's Journal of Health is prepared to testify that millions of teeth which will be lost this year, might be saved for life, by proper care. We define "proper care" as being, in the main, perfect cleanliness and an avoidance of injurious substances. We *know* that perfect cleanliness can be secured by the daily use of a good, sound, stiff brush, charged with a cleansing, antiseptic dentifrice, and passed up and down through the teeth and not simply across their surfaces. We know that Lyon's Tooth Tablets, of which we have often spoken, entirely fulfill the conditions of the true dentifrice. We urge their use ; we insist upon their use in our own family and among our friends. They are grateful to every sense ; harmless in their effect upon the mouth and teeth, and convenient, cleanly and economical in use.

